# Supplementary material for: Navigating antiretroviral adherence in boarding secondary schools in Nairobi, Kenya: A qualitative study of adolescents living with HIV, their caregivers and school nurses
Source: PLOS Glob Public Health. 2023 Sep 25;3(9):e0002418. doi: 10.1371/journal.pgph.0002418 (PMC10519593; doi:10.1371/journal.pgph.0002418)
Supplement: S1 Codebook — (PDF) [file pgph.0002418.s002.pdf]

Codebook Adolescent FGD

Nickname	Name	Description
A	Adolescent experience with the boarding life	General experience within the school settings
Aa	Disclosure of HIV status	Statement on how adolescent disclose their HIV status to friend, teachers and school nurses, and approaches used
Ab	Experience with school search protocols	Statement on the adolescent experience with school search protocol at gate and dormitory in relation to storage or carrying antiretroviral drugs
Ac	Barrier to HIV disclosure at schools	Reasons for non-disclosure of HIV status to either school nurse or a teacher
Ad	Reminder strategies	Strategies used by adolescent to remember to take antiretroviral.
Ae	Medication taking	Statement on time and how adolescent take their antiretroviral drugs
Af	Peers discussion on HIV	Statement on comfortability to discuss topical issues among peer e.g. on HIV ,sex etc.
Ag	Reasons for skip drugs	Statement on the reason for skips drugs while at school
D	Views on school based counselling	Statement on what adolescent like or dislike with regard to introduction of school based counselling session for ALHIV
E	Recommendation	Suggestions on what is needed to improve school support for ALHIV
F	Adolescent –school nurse interaction	Statement on how adolescent interact with their school nurse
G	Like and dislike about school nurse	Statement on what adolescent like about school nurse or dislike
