# Supplementary material for: Navigating antiretroviral adherence in boarding secondary schools in Nairobi, Kenya: A qualitative study of adolescents living with HIV, their caregivers and school nurses
Source: PLOS Glob Public Health. 2023 Sep 25;3(9):e0002418. doi: 10.1371/journal.pgph.0002418 (PMC10519593; doi:10.1371/journal.pgph.0002418)
Supplement: S2 Codebook — (PDF) [file pgph.0002418.s003.pdf]

Codebook School nurse IDI

Nickname	Name	Description
A	School nurse experience in school clinic	General view on school nurses with offering care to students
Aa	Available support for ALHIV	Statement on support available for ALHIV eg drugs storage, medication refill reminder
Ab	Barriers to HIV disclosure at school	Statement on what are barrier to HIV disclosure to school nurses among adolescents
Ac	Support acceptance among adolescent	Statement on why adolescents are likely to accept adherence support while at school
Ad	Training and supervision need	HIV/AIDS-related assessment on the training and supervision need
Ae	Approach to HIV disclosure at school	Statement on how caregivers and adolescents disclose their HIV status to school nurses
Af	Challenges in school	Challenges experienced by school nurse at school
B	Peer support	Statement on any peer support activities within the school
C	Recommendation	Suggestions on what is needed to improve the support for ALHIV
