# Supplementary material for: Navigating antiretroviral adherence in boarding secondary schools in Nairobi, Kenya: A qualitative study of adolescents living with HIV, their caregivers and school nurses
Source: PLOS Glob Public Health. 2023 Sep 25;3(9):e0002418. doi: 10.1371/journal.pgph.0002418 (PMC10519593; doi:10.1371/journal.pgph.0002418)
Supplement: S3 Codebook — (PDF) [file pgph.0002418.s004.pdf]

Codebook-Caregivers FGD

Nickname	Name	Description
A	Caregiver support	General view on how caregiver support their adolescent
Aa	Reminders strategies at home	Reminder strategies employed by caregiver at home to enhance adherence
Ab	Adolescent coping strategies	Statement on how adolescents are coping with antiretroviral adherence while in school
B	Caregiver views on school nurse	Statement on what they like or dislike about school nurses
C	School related challenges	Statement on challenges encounter by adolescent while in school
D	Recommendation on how facility staff can support school nurses	Statement on ways facility staff can support school nurses in enhancing adolescent adherence in school
E	Disclosure to school nurses	Statement on how caregivers facilitate HIV disclosure to school nurses
Ea	Reason why adolescent decline to disclose to school nurses or teachers	Reason provided by caregivers on why adolescence are reluctant to disclose their HIV status in school setting
Ec	Barrier to disclosure at school	Statement on existing barrier to HIV disclosure at school
Eb	Approaches to disclosure in school	Statement on caregiver approach to facilitating HIV disclosure in the school setting
F	Ongoing support at school	Statement on already support available to adolescent in boarding school
G	General concerns raised	General concerns raised by caregivers in relation to introduction of school based adherence e.g. confidentiality ,stigma
