# Supplementary material for: Navigating antiretroviral adherence in boarding secondary schools in Nairobi, Kenya: A qualitative study of adolescents living with HIV, their caregivers and school nurses
Source: PLOS Glob Public Health. 2023 Sep 25;3(9):e0002418. doi: 10.1371/journal.pgph.0002418 (PMC10519593; doi:10.1371/journal.pgph.0002418)
Supplement: S1 Text — (PDF) [file pgph.0002418.s005.pdf]

Focus Group Discussion Topic Guide – Adolescents

Title: Improving Antiretroviral treatment outcomes among adolescents in boarding schools through school nurse training enhanced adherence counseling

Protocol Version 2.0
Date 8 April 2021

Interviewer instructions:

**0.0 Interview Information**<br>***Fill out items A through F before starting the interview.***
(a) Informed assents have been administered: YES / NO<br>*If a consent form has not been signed by each participant, the FGD must not proceed.*<br><br>(b) Focus Group ID: ____________________________<br><br>(c) Date of FGD: ____/_____/_______<br>*Format: DD/MM/YYYY*<br><br>(d) Location of FGD: ____<br><br>(e) Facilitator’s full name: ____<br><br>(f) FGD start time: ____________________<br>*Format: HH:MM am or pm*<br><br>(g) FGD end time: ____________________<br>*Format: HH:MM am or pm*

Facilitator introduction: [DO NOT READ; GUIDE ONLY]

Hello. My name is _______, and I am working at _______. Thank you for taking the time to talk with me today.

The purpose of this FGD is to understand your experiences while at school, your perceptions of school-based counseling services, benefits, and barriers.

There are no right or wrong answers to these questions. People have different views, and we are interested to learn more about these experiences from you. Today, I am here to learn from you since you are an expert in your own life experiences and opinions.

This FGD should take around an hour to complete. Please let me know if at any time you questions have, if something I say is not clear, or if you need to take a break.

Part 1. Adolescents’ health care seeking in boarding schools

As I mentioned, the goal of this work is to understand the experience and challenges encountered by students living with HIV while at school. I’d like to start today by just getting to know a bit about your school life.

1.1 Can you tell me a bit about students’ life in boarding school?

- What time do students in your school wake up, take breakfast, and sleep?

1.2 We understand that students living with HIV require specific needs (e.g., psychosocial support) while in boarding school.

- For the students living with HIV and are learning in boarding schools, what are their options for storing their medication?
- When do they have the best time and privacy to take their medication?
- Do they have any trusted friends whom they have disclosed their HIV status to? If yes, how did they identify those friends?
- What motivated them to share their HIV status with their friends?
- Do they feel comfortable discussing with their friends about sex, adherence to treatment, and other issues affecting their health? Why or why not?
- How satisfied are they with the support they receive from their friends, If any?

1.3 Now I would like us to discuss about students’ experience with getting health care while in boarding school.

- How often does a student visit the school nurse in a term? If yes, what kind of medical issues do they usually have?
- Do students living with HIV disclose their HIV status to the school nurse, teacher, or matron? If yes, how do they go about it?
- What motivates these students to disclose their HIV status to the school nurse, teacher or matron?

1.4 What are some of the thing’s students find easy to discuss with the school nurse? What are some of the things they find more challenging to discuss?

- How easy or difficult is it for students living with HIV to disclose their HIV status to the school nurse, teacher or matron?
- What reasons would make students living with HIV not want to disclose their HIV status to the school nurse, teacher or matron?
- What do you think should be changed about the health care provided for students living with HIV in boarding schools?

Part 2. Adolescents living with HIV psychosocial experiences at school

Now I'd like to transition to talking more about counseling services. Students living with HIV undergo a lot of challenges while at boarding school and this can result in them feeling depressed, stressed, or worried. I would like to hear about your experiences, if any, in receiving psychosocial support from the school nurse.

2.1 We'd like to understand more about students' adherence to medication while in boarding school.

- How well do you think students take their medication while in school?
- What are some of the reasons sometimes students skip taking their drugs?
- Who or what helps them remember to take their drugs?
- What do you think could be done to help students take their drugs better?
- What support can school administration and school nurses provide to help students with their treatment, if any?

2.2 Describe to me some experiences students have with the school nurse while seeking health care services.

- How do school nurses respond to or treat students living with HIV?
- What do students like most about the school nurse? What do the students dislike most about the school nurse?
- What things would you like changed/ not changed about the school nurse, if any?

Part 3. Perceptions of providing psychosocial support /counseling

For this final part of the interview, I'd like to talk about feelings and perceptions on receiving counseling support while at school.

3.1 What concerns do students living with HIV have about receiving psychosocial support services at school?

- Do the students have any concerns about receiving psychosocial services from school nurses or a matron? Tell me more about these.
- Do you think the school clinic is well equipped for providing psychosocial support to students?
- Do you think school nurses should be trained on how to support students living with HIV?
- How often do you think school nurses should offer psychosocial support to students at school?

We have concluded the topics I had prepared to discuss today. Do you have any other thoughts that you would like to share? Do you have any questions for me before we

conclude?

THANK YOU FOR YOUR TIME!

[Mark FGD end time on page 1 (Item F).]
