# Supplementary material for: Navigating antiretroviral adherence in boarding secondary schools in Nairobi, Kenya: A qualitative study of adolescents living with HIV, their caregivers and school nurses
Source: PLOS Glob Public Health. 2023 Sep 25;3(9):e0002418. doi: 10.1371/journal.pgph.0002418 (PMC10519593; doi:10.1371/journal.pgph.0002418)
Supplement: S2 Text — (PDF) [file pgph.0002418.s006.pdf]

FGD Topic Guide –Caregivers

Title: Improving Antiretroviral treatment outcomes among adolescents in boarding schools through school nurse training enhanced adherence counseling

Protocol Version 3.0
Date 7 March 2022

Interviewer instructions: Administer informed consent. Once signed, begin the guide.

**0.0 Interview Information**<br>***Fill out items A through F before starting the interview.***
(a) Informed consent has been administered: YES / NO<br>*If a consent form has not been signed by each participant, the FGD must not proceed.*<br><br>(b) FGD ID: ______________________________<br>*Format: Interviewee Type-MMDDYY-Number of interviews conducted that day where "CG" = Caregiver (e.g., "CG-021222-02" represents the second Caregiver interviewed on 12 Feb 2022)*<br><br>(c) Date of FGD: ____/_____/_______<br>*Format: DD/MM/YYYY*<br><br>(d) Location of FGD: ____<br><br>(e) Facilitator's full name: ____<br><br>(f) FGD start time: ____________________<br>*Format: HH: MM am or pm*<br><br>(g) FGD end time: _____________________<br>*Format: HH: MM am or pm*

Facilitator introduction: [DO NOT READ; GUIDE ONLY]

Hello. My name is _______, and I am working at _______. Thank you for taking the time to talk with me today.

The purpose of this focus group discussion is to understand your experiences in supporting adolescents living with HIV, your perceptions on school counseling services, benefits, and barriers.

There are no right or wrong answers to these questions. People have different views, and we are interested to learn more about these experiences from you. Today, you are in the role of a teacher and I am here to learn from you since you are an expert in your own life experiences and opinions.

This FGD should take around two hours to complete. Please let me know if at any time you a question have, if something I say is not clear, or if you need to take a break. Before we start, do you have any questions?

**Part 1. Basic information about the participant**
As I mentioned, the goal of this work is to understand how best to support adolescents living with HIV while in school. I'd like to start today by just getting to know about how you support your children while in school.<br><br>**Ice breaker**<br><br>Now I will like each one of you to describe the work you do, where are you working, and the community where you live.<br><br>***1.1 Can you tell me a bit about your community?***<br>• How do people in your community interact with one another?<br>• How do people in your community show support for one another? If someone requires help who do they go to?<br><br>***1.2 HIV related stigma in the community and its influence on HIV preventive behaviors.***<br><br>• What do people in the community think or say about people living with HIV?<br>• Do you feel comfortable discussing with adolescents about their sexual behavior, mental health, and HIV prevention and treatment? Why or why not?<br><br>***1.2 Adolescents living with HIV are often a unique population with specific needs around HIV. What has your experience been like in meeting those needs?***<br>• What role do caregivers play in HIV prevention, and treatment adherence?
**Part 2. Perception on school nurse counseling services**
Now I'd like to transition talking more about counseling services. Adolescent living with HIV experiences a lot of challenges while at school and this can result in them feeling depressed, stressed, or worried. I would like to hear about your perceptions if any about the role of school nurses or matrons in supporting these adolescents.<br><br>***2.1 We'd like to understand more about adherence to treatment among adolescents living with HIV while in school.***<br>• What do adolescents say about how they take their drugs while in school? How easy or difficult is it for them to take their drugs?<br>• What challenges contribute to them skipping their medicine?<br>• How do you think some of these challenges can be best addressed?<br>• Do you think adolescent should be encouraged to disclose their status? Why, whom should they disclose to while in school?<br>• What concerns do you have about school nurses or the matron? Confidentiality, stigma?<br>• How can confidentiality and stigma be address in school?

Part 3. Acceptability of school counseling services

For this final part of the interview, I'd like us to talk about your acceptability of school-based counseling services for adolescents living with HIV.

3.1 What might you like or dislike about school-based counseling if it was to be introduced for adolescents living with HIV?

- Do you think the schools are well equipped for providing psychosocial support to adolescents?
- Do you think school nurses are confidential?
- Do you think if school nurses were trained, they will be in a better position to support these adolescents? If yes, why? If not, why not?
- Why do you think some caregivers are reluctant to seek support from school nurses or the matron?
- How can caregivers and adolescents make their HIV status known to the school nurse or matron, if any? When is the best time to disclose HIV status to school nurses?
- How important do you think it is for adolescents to disclose their HIV status to the school nurse or matron?
- Do you feel that school nurses bring benefits to these adolescents in adhering to treatment? Why or why not?
- In what ways could the school administration and school nurses provide psychosocial support to these adolescents?

We have concluded the topics I had prepared to discuss today. Do you have any other thoughts that you would like to share? Do you have any questions for me before we conclude?

THANK YOU FOR YOUR TIME!

[Mark interview end time on page 1 (Item F).]
