# Supplementary material for: Navigating antiretroviral adherence in boarding secondary schools in Nairobi, Kenya: A qualitative study of adolescents living with HIV, their caregivers and school nurses
Source: PLOS Glob Public Health. 2023 Sep 25;3(9):e0002418. doi: 10.1371/journal.pgph.0002418 (PMC10519593; doi:10.1371/journal.pgph.0002418)
Supplement: S3 Text — (PDF) [file pgph.0002418.s007.pdf]

IDI guide –school nurse

Title: Improving Antiretroviral treatment outcomes among adolescents in boarding schools through school nurse training enhanced adherence counseling

Protocol Version 3.0
Date 20th April 2022

Interviewer instructions: Administer informed consent. Once signed, begin the guide.

**0.0 Interview Information**<br>***Fill out items A through F prior to starting interview.***
(a) Informed consent has been administered: YES / NO<br>*If consent form has not been signed by participant, interview must not proceed.*<br><br>(b) Interview ID: ____________________________<br>*Format: Interviewee Type-MMDDYY-Number of interviews conducted that day where "SNP" = School Nurse Provider (e.g., "SNP-021222-02" represents the second Healthcare Provider interviewed on 12 Feb 2022)*<br><br>(c) Date of interview: ____/_____/_______<br>*Format: DD/MM/YYYY*<br><br>(d) Location of interview: ____<br><br>(e) Interviewer's full name: _______________________________________<br><br>(f) Interviews start time: ____________________<br>*Format: HH:MM am or pm*<br><br>(g) Interview end time: _____________________<br>*Format: HH:MM am or pm*

Facilitator introduction: [DO NOT READ; GUIDE ONLY]

Hello. My name is _______, and I am working at _______. Thank you for taking the time to talk with me today.

The purpose of this IDI is to understand your experiences in supporting adolescents living with HIV, your perceptions on school counseling services, benefits, and barriers.

There are no right or wrong answers to these questions. People have different views, and we are interested to learn more about these experiences from you. Today, you are in the role of a teacher

and I am here to learn from you since you are an expert in your own life experiences and opinions.

This IDI should take around one hours to complete. Please let me know if at any time you a question have, if something I say is not clear, or if you need to take a break. Before we start, do you have any questions?

**Part 1. Basic information about the participant**
As I mentioned, the goal of this work is to understand how best to support adolescents living with HIV while in school. I'd like to start today by just getting to know about how you support your children while in school.<br><br>**Ice breaker**<br><br>What's is your experience in providing clinical service at school in general?<br><br>***1.1 Can you tell me a bit about your school?***<br>• How do students in your school interact with one another?<br>• How do students show support for one another? If someone requires help who do they go to?<br>***1.2 Can you tell me a bit about your professional life?***<br><br>• What is your main role in this school?<br>• How long have you worked there?<br>• What does a typical day look like for you?<br>• How often do you see these students at clinic? What other medical services do they come to seek? How long do you typically spend with them on an average clinic visit?<br>• how did you come to learn about HIV status for some these adolescents (if any)?<br><br>***1.3 HIV related stigma in the community and its influence on HIV preventive behaviors.***<br><br>• What do students think or say about people living with HIV?<br>• Do they feel comfortable discussing with adolescents about their sexual behavior, mental health, and HIV prevention and treatment? Why or why not?<br><br>***1.4 What concerns do you have about providing psychosocial support services***<br><br>• Do you have any concerns about providing psychosocial services? Tell me more about these.<br>• Do you think your school are equipped well to support these adolescents in providing psychosocial support?<br><br>***1.5 What (if anything) excites you about providing psychosocial support at school?***

- Would you like to work at an HIV clinic in future and providing HIV service eg counseling? Why or why not?

Part 2. Perception on school nurse counseling services

Now I'd like to transition talking more about counseling services. Adolescent living with HIV experiences a lot of challenges while at school and this can result in them feeling depressed, stressed, or worried. I would like to hear about your perceptions if any about the role of school nurses or matrons in supporting these adolescents.

2.1 We'd like to understand more about adherence to treatment among adolescents living with HIV while in school.

- What do adolescents say about how they take their drugs while in school (if any)? How easy or difficult is it for them to take their drugs?
- What challenges contribute to them skipping their medicine?
- How do you think some of these challenges can be best addressed?
- Do you think adolescent should be encouraged to disclose their status? Why, whom should they disclose to while in school?
- How can confidentiality and stigma be address in school?

2.2 A few years ago, the Kenyan Ministry of Health released Adolescent Package of Care Guidebook. Had you heard anything about this guide?

- IF YES: What had you heard about the guide?
- IF YES: Do you have this guide at your clinic?

2.3 What training or support would you need to provide support and counseling services to adolescent at school clinic?

- What are some core components of this training you e.g example an ideal model of training and school administration support that would help you provide counseling at the clinic?

2.4 What might you like or dislike about school-based counseling program

- Do you think the schools are well equipped for providing psychosocial support to adolescents?
- What challenges do school nurses encounter in supporting ALWHIV?
- What barrier do students face in a school setting for adherence? do you think school nurses have a role to play to address this barrier?
- Why do you think some caregivers are reluctant to seek support from school nurses or the matron?
- How can caregivers and adolescents make their HIV status known to the school nurse or matron, if any? When is the best time to disclose HIV status to school nurses?
- How important do you think it is for adolescents to disclose their HIV status to the school nurse or matron?

- Do you feel that school nurses bring benefits to these adolescents in adhering to treatment? Why or why not?
- In what ways could the school administration and school nurses provide psychosocial support to these adolescents?

We have concluded the topics I had prepared to discuss today. Do you have any other thoughts that you would like to share? Do you have any questions for me before we conclude?

THANK YOU FOR YOUR TIME!

[Mark interview end time on page 1 (Item F).]
