# Supplementary material for: Navigating antiretroviral adherence in boarding secondary schools in Nairobi, Kenya: A qualitative study of adolescents living with HIV, their caregivers and school nurses
Source: PLOS Glob Public Health. 2023 Sep 25;3(9):e0002418. doi: 10.1371/journal.pgph.0002418 (PMC10519593; doi:10.1371/journal.pgph.0002418)
Supplement: Attachment — Submitted filename: Response to Reviewers.docx [file pgph.0002418.s008.docx]

| **Thank you to all the reviewers for your comments, which have greatly strengthened this manuscript, please see below for a detailed response to each question raised.**  **We have also revised out manuscript title from ‘’How adolescents living with HIV navigate antiretroviral adherence in boarding school in Nairobi Kenya: a qualitative study of adolescents, caregivers and school nurses’’ to ‘’Navigating antiretroviral adherence in boarding secondary schools in Nairobi, Kenya: a qualitative study of adolescents living with HIV, their caregivers and school nurses’’** | | |
| --- | --- | --- |
| **ISSUE RAISED** | **RESPONSE** | **Document page number** |
| 1. **EDITOR comments:** a) **Re: data analysis:** "Analysis followed deductive and inductive thematic approaches, with initial categories based on the interview guide coded by NK and emergent themes integrated into the second round of coding by IM." Please provide some more expansive details on qualitative data analysis-was there a theory or model used for analysis? what type of deductive and thematic approach(es) was/were used? Cite any sources applicable. There was no clear indication (per requirements of the COREQ checklist) of methodological orientation underpinning the study | Thank you for your very insightful comments. We have revised this section to include more details on the data analysis process. We did not use a theory or model for analysis as this study design was largely exploratory. We have also included a reference on the thematic data analysis method. The section now reads “Data analysis was conducted using deductive and inductive thematic approaches and followed the steps described by Braun & Clarke, [2006], to facilitate finding of patterns and making meanings of data. Codebooks were developed iteratively, with initial codebooks developed based on FGD and IDI guides and revised using emerging themes from preliminary data. De-identified transcripts were imported into NVivo 12 (QSR International, Australia) [16] for coding. Ten percent of the transcripts were coded by two coders (NK and IM) to ensure interrater reliability and any differences were discussed until agreement was reached. The remaining transcripts were coded independently by NK and IM and integrated any additional emerging themes. Code excerpts were exported into Microsoft Word, and summaries written to capture the key themes from each code, focusing on topics related to facilitators and barriers to school-based adherence support from adolescent, caregiver, and school nurse perspectives.” | See tracked manuscript page 7 (line number 124 &135) |
| b) Related to the above, the pages cited in the COREQ checklist do not appear to correlate with the main manuscript. I take it those are line numbers and not page numbers, if so, please indicate in the COREQ checklist. Also correct the first "page number" listed as "2rd" for interviewer/facilitator. | We apologize for apologies for the mix-up. We have now used page numbers and corrected the first page. |  |
| c) **Section titled** "Adolescents Boarding School Experience" should really indicate that this was adolescents *living with HIV*, otherwise it reads like a general description of adolescents in boarding school. Suggest "Boarding School Experience for Adolescents Living with HIV." Same comment for "Adolescent" section in Table 1. It should be ALHIV (expand the term if not defined as a table footnote) | Thanks for you for pointing out this. As suggested by the reviewer, we have revised to read ‘Boarding school experience for adolescents living with HIV’ | See tracked manuscript page 8 &12 (line number 152 &226) |
| i) **Discussion**, line 367: Other researchers *have* (not HAS) found....Please review the manuscript thoroughly for grammar and syntax errors. It may be good to have an author or non-author with strong English editing skills to review so that none of these errors are missed. | We apologize for the error. We have corrected this and reviewed entire manuscript to ensure that grammatical errors are corrected. | See tracked manuscript page 20 (line number 448); all the other pages. |
| 2. **Journal Requirements:** a) Please review your reference list to ensure that it is complete and correct. If you have cited papers that have been retracted, please include the rationale for doing so in the manuscript text, or remove these references and replace them with relevant current references. Any changes to the reference list should be mentioned in the rebuttal letter that accompanies your revised manuscript. If you need to cite a retracted article, indicate the article’s retracted status in the References list and also include a citation and full reference for the retraction notice | Thank you for this important comment, we have reviewed the reference provided and to our best of our knowledge all references are complete and correct. | See tracked manuscript page 25 |
| b) We have noticed that you have uploaded Supporting Information files, but you have not included a list of legends. Please add a full list of legends for your Supporting Information files after the references list. | Thank you for this important comment, we have attached list of legend |  |
| c) In the online submission form, you indicated that "De-identified participant data, including interview transcripts, will be made freely available after publication upon request to the corresponding author and with concurrence of the KEMRI Scientific and Ethics Review Unit". All PLOS journals now require all data underlying the findings described in their manuscript to be freely available to other researchers, either 1. In a public repository, 2. Within the manuscript itself, or 3. Uploaded as supplementary information. This policy applies to all data except where public deposition would breach compliance with the protocol approved by your research ethics board. If your data cannot be made publicly available for ethical or legal reasons (e.g., public availability would compromise patient privacy), please explain your reasons by return email and your exemption request will be escalated to the editor for approval. Your exemption request will be handled independently and will not hold up the peer review process, but will need to be resolved should your manuscript be accepted for publication. One of the Editorial team will then be in touch if there are any issues | Thank you for this important comment. We have corresponded with the editor and have received a waiver for sharing the complete interview transcripts. Considerable privacy concerns exist regarding full length interviews done with minors living with HIV.  We are sharing codebook for caregiver FGD, adolescent FGD and school nurse IDI and their corresponding interview guide named (Supplement File 1 to 6.  We have revised this statement to read ‘’The semi-structured interview guide and the codebook used to collect and analyze the qualitative interviews are included as additional files. The full transcripts of the qualitative interviews may be available after consultation with the KEMRI Scientific and Ethics Review Unit, as per the ethical approval obtained for this research.”  This position is consistent with other researchers, who have noted a number of epistemological, methodological, legal, and ethical concerns regarding open data in qualitative research. These concerns include debate over privacy issues as well as secondary analysis, which entails the transparent reuse of existing qualitative data from prior research studies in order to validate conclusions and/or produce novel insights from old data. Reference; [https://journals.sagepub.com/doi/10.1177/1609406918823863](https://urldefense.com/v3/__https:/journals.sagepub.com/doi/10.1177/1609406918823863__;!!K-Hz7m0Vt54!hqLxLwHA2If2pkZAXwNwYKfJJw7RVyTAmQ7YqKbqms65fuuxKULUu_v-WVYpQ3WyJFfG-Mj-TUU9ZdktwOIcJPOo0g$)'' | Attached as a separate file |
| 3) **Reviewer #1:** A) LINE 2-3: Consider revising this statement to refer to boarding secondary schools rather than secondary schools. This is because being a student in day secondary schools may not have a directly affect ART adherence. | Thank you for this important comment. As suggested, we have revised to read “boarding secondary school”. | See tracked manuscript page 3 (line number 2) |
| B) LINE 14: The word some ALHIV does not give readers an idea of the proportion who disclosed. Perhaps rewording to reflect a rough estimate using words such as few, most, majority etc. | Thank you for this important comment, we have revised it to read “Most of the ALHIV reported having disclosed their HIV status to a school nurse or teacher during admission.” | See tracked manuscript page 3 (line number 14) |
| C) LINE 47-48: It would enrich the paper to include information indicating the estimated number/proportion of ALHIV attending boarding schools if such data is available. This would showcase the magnitude of the problem and potential impact of the suggested interventions among ALHIV. | Thank you for this important comment, unfortunately there is no data available on proportion of ALHIV in boarding school. However, we have indicated in that ‘’In Kenya, there are about 4300 boarding secondary schools nationally, and of these, approximate 50% are public boarding schools. According to the Kenya Population-based HIV Impact Assessment (KENPHIA) 2018 report, children were among those affected by the HIV burden, with about 139,000 children aged 0-14 years living with HIV in Kenya’’. | See tracked manuscript page 4 (line number 49-53) |
| D) LINE 62: If there was more than one nurse per level, how was recruitment for participation in IDI done? Provide more information on the participants’ selection process | Thank you for this important comment, we have reworded the sentence to read, “For IDIs, we only recruited school nurses employed in Nairobi County boarding schools either in urban or peri-urban settings. The boarding schools were categorized into national, county, and sub-county levels. The boarding school selection was based on their location and proximity to the facility, and we prioritized closer boarding schools with the aim of recruiting at least 2 school nurses per level, having obtained prior school leadership permission. The inclusion criteria for school nurses included at least 1 year of boarding school experience and willingness to provide consent. There were no exclusion criteria.” | See tracked manuscript page 5 (line number 79-84) |
| E) LINE 63-64: Any exclusion criteria? If there was, it is important to mention | Thank for your comment, we have indicated that: “There were no exclusion criteria’’. | See tracked manuscript page 5 (line number 84) |
| F) LINE 71-72: The study setting is not clearly described. There is need to provide overview of the demographic, psychosocial and other relevant characteristics of the settings, especially as it relates to potential stigma and discrimination of adolescents living with HIV, procedures for access to available HIV screening, treatment and counselling care services, cadre and level of experience and relevant training exposure of healthcare workers at the facility and schools | Thank you for your comment. We reworded this, and it now reads: “We recruited adolescents and caregivers from Pumwani Hospital, and school nurses from boarding secondary schools within Nairobi County. Pumwani Hospital is one of the largest public facilities in Kenya serving a population of about 29000 and is located in a low-income urban area in Nairobi. The facility has an outpatient HIV clinic which supports HIV counseling and testing, prevention-of-mother-to-child-transmission, care and treatment, and virology laboratory services. The clinic serves about 370 adolescents living with HIV, about 80 of whom are enrolled in various boarding schools. Adolescent tailored services such as ART adherence, appointment keeping, sexual reproductive health, and mental health are offered on monthly basis in addition to adolescent day activities, universally referred as to Operation Triple Zero (OTZ) fun days, held quarterly [18]. The events focus on empowering adolescents and young people living with HIV to commit to ‘’triple zero outcomes’’: zero missed appointments, zero missed drugs, and zero viral load. The HIV clinic services are offered by clinical officers, nurses, counselors and peer mentors.”  In addition, all school nurses had diploma certificate in nursing and we have listed their experience in the table 1 | See tracked manuscript page 5 (line number 60-73) |
| G) LINE 81-83: Does this statement imply that EO and EA were undergraduates and female scientists? ‘EO and EA, BA undergraduate, female social scientists with 2-5 years of experience in qualitative research facilitated FGDs among caregivers and adolescents with help of a research assistant’. Considering that the entire data collection for FGD and IDI were managed by undergraduates despite their 2-5 years of experience, was there any supervisory oversight by potentially more experienced authors NK, ACR and KA, especially to ensure that developed guides were utilized appropriately as designed? | Thank you again for this important comments. For clarity purposes we have reworded the statement to read; “The interview guides were developed by the investigators (NK, ACR, and KA) based on the topic of interest with additional inputs from a social scientist (EO). The guides were piloted among the caregivers and adolescents attending care and treatment in a different health facility prior to conducting the study. The FGDs were conducted by EO and EA, female social scientists, with Bachelor of Education and Bachelor of Arts degrees, and 2-5 years of experience in qualitative research methods, with the help of a research assistant (SK). IDIs were conducted remotely via phone depending on the school nurses’ availability. The relative youth of the social science investigators was considered an advantage for working with teens during this project and both had prior experience in administering FGDs and IDIs. NK, ACR, and KA supported FGD and IDI guide development and provided supervisory oversight during data collection, coding, and analysis’’. | See tracked manuscript page 6 (line number 108-118) |
| H) LINE 85-86: In view of sensitivity of the topic, did the facility staff members use any criteria to identify interested caregivers and adolescents, especially while maintaining privacy, confidentiality and voluntary participation? For instance, did the facility staff have prior knowledge of the adolescents’ personalities and potential disposition towards participating in FGDs? It would be good to see how many did not consent, as well as rationale for non-consenting. Lessons may be useful for future research/researchers in similar settings. | Thank you for your comment. Below is our response to these comment.  The facility staff line listed all adolescents living with HIV in boarding schools using facility registers and contacted their caregivers via a phone call. Only caregivers who were reached via phone call were given information on the purpose of the study, procedures, risk and benefits, and the scheduled FGDs dates. All caregivers were invited a week earlier and offered FGDs attendance options based on their interest and availability. The HIV clinic nurses had no influence towards adolescent participating in the FGDs, as FGDs were conducted during adolescent fun day at the facility and all those who were present were offered a chance. Most of the adolescent were familiar to each other and this could be attributed to their active participations in fun day activities. The few adolescents who declined to participate in the FGDs were recent transfer in or newly diagnosed. | See tracked manuscript page 7 (line number 121-127), page 8 (line number 169-176) |
| I) LINE 114-120: Though there was disproportionately higher number of girls-only compared with boys-only schools (13 vs 3), there were more male than female participants (10 vs 8). The rationale for this would be good to know. Was it that more girls than boys declined consent to participate? This distribution is not clear, considering that 13 consenting girl-only schools were visited and there were only 8 female participants presented here. Kindly clarify. Also, in table 1, thought the numbers are small, it would still be good to include the proportions/percentages alongside the numbers in the last column. This will aid comparison across subgroups. | Thank you for this comment. It was not purposive to have a higher representation of girls secondary schools. As described, the 13 girls and 3 boys boarding secondary schools visited were only for recruiting school nurses to participate in the IDIs. The schools were selected as described in Methods lines 80-85.  The boys and girls who participated in the FGDs were recruited during adolescent fun day activities at the facility clinic during school holidays, regardless of the boarding school they came from in Kenya. Therefore, we did not visit any boarding school to recruit adolescents to participate in the FGD, as this would have breached their HIV status confidentiality. There were no difference in girls and boys who declined to consent for participation; however, more boys were in boarding school compared to girls. Thus majority of boys met our inclusion criteria and participated in the FGD.  We have included percentage alongside the number as outline in table1: *Characteristics of participants*. | See tracked manuscript page 5 (line number 80-85),  page 8 (line number 150-153)  page 8 (line number 157) |
| J) LINE 120: Not all potential audience (settings) would understand Form in the context of the ALHIV's class as it may not be a universally known terminology. Consider adding in bracket alternate meaning of form e.g grade equivalent | Thank you for this important comment, we have provided an equivalent grade as form 1, 2, 3, 4 be of equivalent for grade 9, 10, 11 and 12 in Table 1: Characteristics of participants | See tracked manuscript page 8 (line number 153) |
| K) LINE 163-165; 170-173: These lines provide commendable attempt at balance of expression of strategies for (maintaining) non-disclosure of HIV status. However, these are strategies utilized by the school nurse/management only. It would be good to see expressions of non-disclosure strategies employed by the adolescents, especially while they live and interact with their peers. Also, if it is extractable from the interviews, it would be good to see verbal and non-verbal cues that suggest degree of confidentiality, privacy, peer interaction and other relevant perceived psychosocial effects of HIV status disclosure as well as non-disclosure especially among the adolescent participants. | Thank you for this comments, we have shared some of the non-disclosure strategies employed by adolescent while in school under sub-heading, ***Coping strategies in school setting, Peer-to-peer discussions about HIV and sex at school, Peer-to-peer support in boarding school***  For example ‘’ *‘’I take mine in the morning only and I lie that it is an allergy medication. I also changed the storage bottle; my mum changed it for me so even if they see the bottle, they will just think it is medication for my chest.’’* (18-year-old female adolescent R3, FGD1)  *‘’I would rather pretend that I don’t know than join in the discussion because they will start looking at me like I know too much information.’’*(16-year-old male adolescent R2, FGD-1)  “*I feel comfortable because I pretend that I am like them*” (18-year-old female adolescent R8, FGD1) | See tracked manuscript page 13 (line number 263 onwards) |
| L) LINE 190: Consider qualifying this statement in the context of antiretroviral treatment. “Adolescents’ boarding school experience” may be too broad. | Thank you for this important comment, we have revised to “boarding school experience for adolescents living with HIV.” | See tracked manuscript page 12 (line number 231) |
| M) LINE 406-408: Was there a difference in opinions and/or strategies between female and male ALHIV? If there was consider reporting these in your result/discussion. | Thank you for this important comment, there were no notable difference in the opinion. However we have added additional code under peer-peer discussion.  “*I feel comfortable because I pretend that I am like them*” (18-year-old female adolescent R8, FGD1) | See tracked manuscript page 14 (line number 290) |
| N) LINE 457: This statement may be true but in the result section, no quotes were mentioned to showcase the forms of stigma experienced/reported by the ALHIV participants. Most of the quotes pointed to the fear of stigma rather than stigma experienced. Consider bringing out quotes that showcase experiences of stigma if there were. | Thank you for this important comment, we have included additional quotes under subhead, **Boarding school experience of adolescents living with HIV**. In addition, we interpreted fear of stigma as self-stigma  *‘’When I was in form 1, I told my friend called [A] who later transferred out. I trusted her and told her that I was HIV positive and after 1 week, I started hearing rumors about it. I cried a lot and went to the deputy. The deputy talked to her and even gave her some punishment’’.* (17-year-old female adolescent R4, FGD)  *‘’For example with these guidance and counselling teachers, as soon as you leave that room, you become the topic of discussion. What you disclose to that teacher, she will disclose to other teachers and so in class, there is a way the teacher looks at you. These teachers like backbiting people’’* (18-year-old female adolescent R7, FGD) | See tracked manuscript page 13 (line number 247-254) |
| 4) **Reviewer #2:** A) There is no citation in text for the reference number 3 which goes to explain why references for Kimera et al and Skerritt et al are one reference away from those cited in text. | We apologize for this omission. We have provided reference | See tracked manuscript page 4 (line number 30) |
| B) Secondary reference for global ALHIV data in a paper from Togo; certainly there are UNAIDs/WHO data that can be referenced. | Thank for your suggestion; we have now reference UNICEF data 2020 | See tracked manuscript page 4 (line number 30) |
| C) Though references made to teachers being facilitators/barriers to adherence etc e.g. in lines 361 and 387, any reason why their views were not sought? | Thank for your comments. While we acknowledge that teachers are important in the academic lives of students, unfortunately we did not have ethical approval to interview teachers as we assumed counseling was in the docket of nurses. This study has revealed that teachers are also important and any study following on from this one will include teachers. |  |
| D) Line 20- harmonization of terms. "adolescents" rather than "teens" | Thank you for the correction. We have harmonized | See tracked manuscript page 3 (line number 21) |
| E) Line 276 - ...why am [sic]..... | Thank you for the correction, we have revised | See tracked manuscript page 17 (line number 358) |
| F) Line 288- "...students" instead of "...student" | Thank you for the correction, we have revised | See tracked manuscript page 17 (line number 368) |
| G) Line 313- "...faring" instead of "..fairing" | Thank you for the correction, we have revised | See tracked manuscript page 18 (line number 395) |
| H) Line 383- "..another study" instead of "other study" | Thank you for the correction, we have revised | See tracked manuscript page 21 (line number 470) |
