# Supplementary material for: Navigating antiretroviral adherence in boarding secondary schools in Nairobi, Kenya: A qualitative study of adolescents living with HIV, their caregivers and school nurses
Source: PLOS Glob Public Health. 2023 Sep 25;3(9):e0002418. doi: 10.1371/journal.pgph.0002418 (PMC10519593; doi:10.1371/journal.pgph.0002418)
Supplement: Attachment — Submitted filename: Response to Reviewer 2.docx [file pgph.0002418.s009.docx]

| **Thank you to the reviewer for your comments, which have greatly strengthened this manuscript, please see below for a detailed response to each question raised.** | | |
| --- | --- | --- |
| **ISSUE RAISED** | **RESPONSE** | **Document page number** |
| **Reviewer #2:**   A) Could the authors give any comment as to the source of HIV in these clients? A pervasive assumption is that they acquired HIV vertically. If so were the concerns of Mothers in FGDs also a revelation of concern that their status were to be disclosed with school based interventions? | Thank you for this important comment, unfortunately we did not abstract data or explore on source of HIV in the research participants as this was outside the scope of our research. It is interesting that this issue was not raised in the FGDs. |  |
| B) Line 16 " school nurse friendliness, being understanding, fair and confidential" not '..fairness, | Thank you for the correction, we have revised. | See tracked manuscript page 3 (line number 16) |
| C) Line 51 should read approximately 50% not approximate 50% | Thank you for the correction, we have revised | See tracked manuscript page 4 (line number 51) |
| D) Line 115-116- can you clarify who considered the youthful age of social science investigators an advantage? was it the participants or the research team? | Thank you for this comments, youthful age of social science investigators refers to research team. It was considered an advantage by the research team to have younger investigators. This project was funded by RHAY (Reducing HIV/AIDS in Youth) and the grant specifically funded youth researchers to get involved in HIV prevention. | See tracked manuscript page 7 (line number 115-117) |
| E) Line 136 - is the term "learner" of particular significance? if not can these adolescents be referred to as "students"? | Thank you for your suggestion, we have adopted the term ‘’students’’ | See tracked manuscript page 7 (line number 136) |
| F) Line 143- does data saturation refer to thematic saturation? | Thank you again for this important comments. Data saturation refer to thematic saturation, we have also indicated in our manuscript. | See tracked manuscript page 7 (line number 143) |
| G) line 170- was any attempt made to find out why 14 of 25 invited guests did not show up? | Thank you for this comment. Unfortunately we did not find out reasons for non-attendance reason, as FGD participation was based on voluntariness. We have also indicated that no reasons were sought on non-attendance in our manuscript | See tracked manuscript page 9 (line number 170) |
| H) Line 323- I believe the sentence should be .."debunk (myths) of HIV" | Thank you for the correction, we have revised | See tracked manuscript page 16 (line number 325) |
| I) Line 519.... these findings (plural form) | Thank you for the correction, we have revised | See tracked manuscript page 23 (line number 524) |
| J) Line 545.... reword the sentence, "despite a lack of approval to interview teachers,......." | Thank you for the correction, we have revised | See tracked manuscript page 24 (line number 549) |
