# Supplementary material for: Navigating antiretroviral adherence in boarding secondary schools in Nairobi, Kenya: A qualitative study of adolescents living with HIV, their caregivers and school nurses
Source: PLOS Glob Public Health. 2023 Sep 25;3(9):e0002418. doi: 10.1371/journal.pgph.0002418 (PMC10519593; doi:10.1371/journal.pgph.0002418)
Supplement: Attachment — Submitted filename: Response to Reviewer 3 .docx [file pgph.0002418.s010.docx]

| **Thank you to the reviewer for your comments, which have greatly strengthened this manuscript, please see below for a detailed response to each question raised.** | | |
| --- | --- | --- |
| **ISSUE RAISED** | **RESPONSE** | **Document page number** |
| **Editors comments:**   A) Kindly add a brief note on Reflexivity (researchers perspective) as well as trustworthiness in the Limitations- which are better titled 'Methodological Considerations' | Thank you for your comment. We have indicated that, ‘’Researchers participating in this study were from different backgrounds e.g. nurse, medical doctor and social scientist, and all acknowledge their bias, own beliefs and judgement systems going into this analysis. Reassuringly, having investigators from mixed backgrounds helped to minimize any bias that would have been driven by a single individual’s background’’. | See tracked manuscript page 24 (line number 547-552) |
| **Reviewer #1:**   A) Line 16 read better as: “School nurse friendliness, being understanding, fair, and confidential were qualities” and not “confidentiality”. | Thank you for the correction, we have revised. | See tracked manuscript page 3 (line number 16) |
| B) Line 115 should read better as “The relative youthfulness of the facilitators rather than “the relative youth of the social scientists”. | Thank you for the correction, we have revised. | See tracked manuscript page 6 (line number 115) |
| C) Line 117 In this statement “experience in administering FGDs and IDIs”, the word administering should be replaced by “facilitating” which is more appropriate. | Thank you for your suggestion, we have adopted the term ‘’facilitating’’ | See tracked manuscript page 7 (line number 117) |
| D) Line 126-128In this statement “All caregivers were invited a week earlier and offered FGD attendance options based on their interest and availability.” The word interest is not quite clear. It may be best to remove “interest” and simply say “based on their availability”. | Thank you for the correction, we have revised to read,’’ All caregivers were invited a week earlier and offered FGD attendance options based on their availability.” | See tracked manuscript page 7 (line number 126) |
